# The role of brachytherapy for margin control in oral tongue squamous cell carcinoma

**DOI:** 10.1186/s40463-020-00467-w

**Published:** 2020-10-14

**Authors:** Ilia Ianovski, Alex M. Mlynarek, Martin J. Black, Boris Bahoric, Khalil Sultanem, Michael P. Hier

**Affiliations:** 1 Department of Otolaryngology – Head & Neck Surgery, Auckland District Health Board, Auckland City Hospital, Auckland, New Zealand; 2 Department of Otolaryngology – Head & Neck Surgery, Jewish General Hospital, McGill University, Montreal, Quebec Canada; 3 Department Radiation Oncology, Jewish General Hospital, McGill University, Montreal, Quebec Canada

**Keywords:** Oral cavity, Radiation therapy, Neck dissection, Squamous cell carcinoma, Tongue cancer, Oral cancer, Brachytherapy, Survival

## Abstract

**Background:**

The aim of this study is to assess the feasibility and effectiveness of using peri-operative brachytherapy (BRTx) for positive/narrow margins present post primary surgical resection of oral tongue squamous cell carcinoma (OTSCC).

**Methods:**

Prospective single-centre study of patients with OTSCC (T1–3, N0–3, M0) treated with resection of primary tumour ± regional nodal resection and intra-operative insertion of BRTx catheters. BRTx was administered twice daily at 40.8Gy/12Fr for ‘Positive’ (≤2 mm) margins, at 34Gy/10Fr for ‘Narrow’ (2.1-5 mm) margins, and not given for ‘Clear’ (> 5 mm) margins over the course of 5–6 days, 3–5 days post operatively.

**Results:**

Out of 55 patients recruited 41 patients (74.6%) were treated with BRTx, as 12 patients had clear margins and 2 patients had unfavourable tumour anatomy for catheter insertion. EBRTx was avoided in 64.3% of patients. Overall Survival (OS) at 3 and 5 years was 75.6 and 59.1% respectively, while Disease Specific Survival (DSS) was 82.3 and 68.6% at 3 and 5 years respectively. Recurrence and survival outcomes were not associated with margin status or the use of or specific dose of BRTx on Cox regression analysis. Acute and late toxicity secondary to BRTx was minimal.

**Conclusions:**

The use of BRTx after primary OTSCC resection with positive/narrow margins ± EBRTx to the neck ± CTx achieves outcomes comparable to traditional treatment of surgery followed by re-resection or EBRTx ± CTx. Morbidity associated with oral cavity EBRTx or secondary resection and reconstruction is thus avoided. Both acute and late toxicity rates are low and compare favourably with other BRTx OTSCC studies.

**Trial registration:**

Retrospectively registered. https://www.mcgill.ca/rcr-rcn/files/rcr-rcn/2017.06.05_rcn_hn.pdf.

**Level of evidence:**

4

**Graphical abstract:**

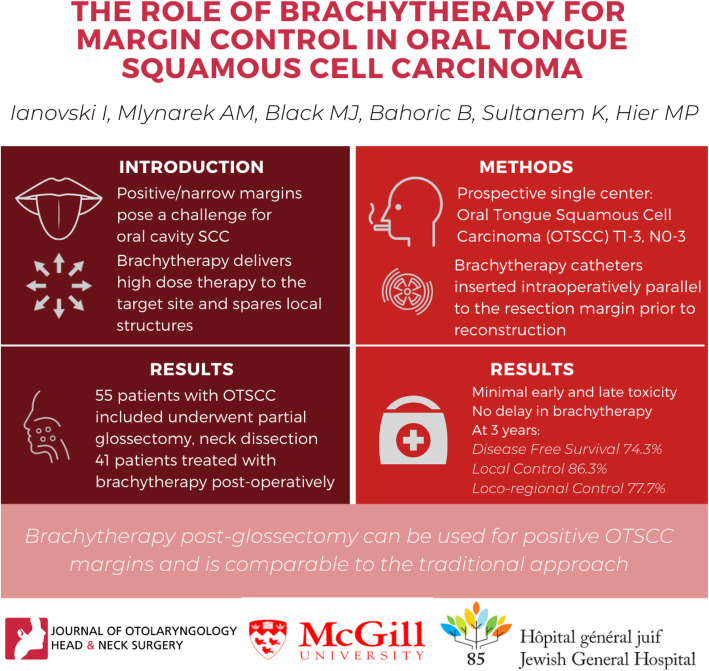

## Background

Oral tongue squamous cell carcinoma (OTSCC) represents approximately 40% of all oral cavity squamous cell carcinoma (OCSCC) [1] and its incidence is increasing internationally [2, 3]. OTSCC is conventionally treated with surgery: wide local excision (WLE) of the primary tumour, elective neck dissection guided by depth of invasion and size of the primary in clinically negative neck (cN0), and therapeutic neck dissection for clinically evident regional metastasis. Adjuvant external beam radiation therapy (EBRTx) ± Chemotherapy (CTx) is given for high risk features: presence of multiple positive regional nodes, extra nodal extension (ENE), Lympho-vascular spread (LVS), peri-neural invasion, and positive/narrow resection margins (< 5 mm).

Adequate resection margins (≥5 mm) remain at the forefront of the ablative surgeon’s aims, as positive/narrow margins are associated with increased risk of local recurrence in Head and Neck cancer [4] and affect survival in OTSCC specifically [5]. Inadequate margins are found in 10–16% of all head and neck cancer cases, despite best surgical attempts [6] and the overall salvage cure rate of recurrent OCSCC has been documented between 21% [7] - 35% [8].

The management of positive/narrow margins poses a challenge to the multi-disciplinary team (MDT). Revision surgery is frequently made difficult due to the use of a free flap or a local flap for the reconstruction of the primary tongue defect, and adjuvant EBRTx is thus recommended. EBRTx for close/positive margins in OTSCC has been shown to improve survival but not to the level of clear margins [5]. EBRTx applied to the oral cavity is associated with significant morbidity, such as mucositis, xerostomia, dysphagia, and osteoradionecrosis (ORN) of the mandible.

Brachytherapy (BRTx) allows for the delivery of high dose conformal radiation to the target site in a shorter time frame, while sparing surrounding normal structures. It has been used as the primary and adjuvant treatment of OTSCC in France, Japan, Czech Republic, South Africa, and Spain [9–14] but there has been no reported North American experience.

The aim of this study was to evaluate the feasibility, local and loco-regional control, survival outcomes, and complications of peri-operative BRTx treatment for positive/narrow margins present post primary surgical resection of OTSCC ± EBRTx to the neck ± CTx depending on pathological indicators.

## Methods

This single-centre prospective project was approved by the Research Ethics Committee of Jewish General Hospital, McGill University. Patients diagnosed with T1–3, N0–3, M0 OTSCC were identified at the multidisciplinary tumour board (MTB) and it was established that the primary tumour was amenable to WLE limited to a partial glossectomy and insertion of BRTx catheters ± surgical resection of ipsilateral/bilateral regional cervical lymph nodes on elective/therapeutic basis. Each patient underwent a thorough clinical examination by at least two members of the surgical team and a single invariable radiation oncologist to assess for the possibility of BRTx. BRTx was not considered feasible in cases where OTSCC came to involve the mandible directly, or was approaching the mandible via floor of mouth (FoM) so as to require BRTx catheters to be placed in direct contact with the bone. The following exclusion criteria were applied: Zubrod performance status > 2, prior EBRTx to the head and neck region, previous CTx, prophylactic use of amifostine or pilocarpine, previous other malignancy except non-melanomatous skin cancer or a carcinoma not of head and neck origin ≤5 years, other treatment for head and neck cancer, active untreated infection. Patients fitting the above criteria were offered to take part in the study and study-specific informed consent form was signed prior to registration.

**Fig. 1:**
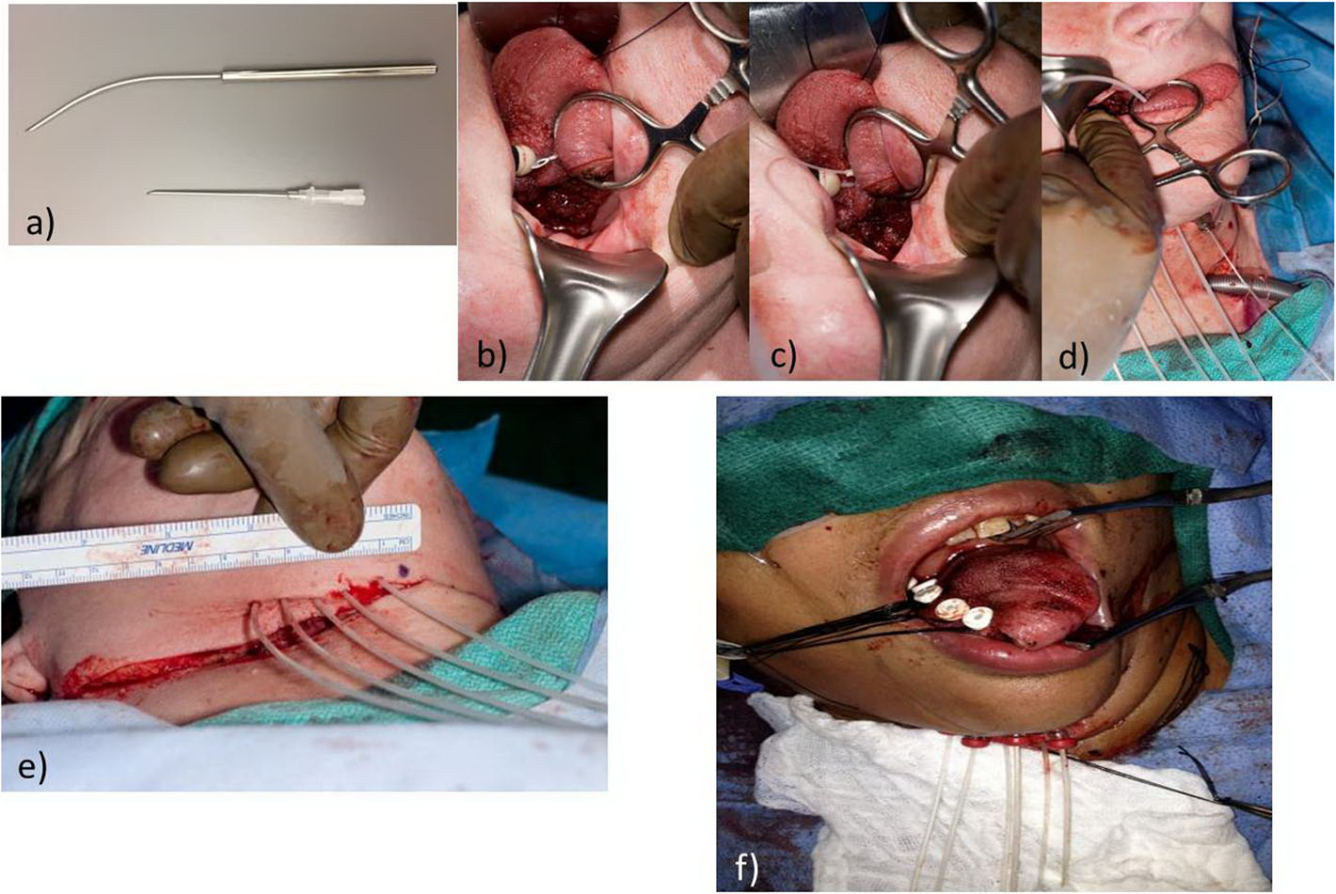
Brachytherapy catheter insertion technique. **a** Stainless steel Brachytherapy catheter insertion guide or 14 Gauge Intra-venous catheter. **b** Catheter guide inserted into residual tongue tissue 3-5 mm from the resection margin. **c** Brachytherapy after-loading catheter passed through the insertion guide. **d** Catheter is passed through the skin and catheter guide removed. **e** Catheters are placed 1–1.5 cm apart and parallel to each other. **f** catheters are secured to skin with semi-lunar buttons. Silk ties placed around the catheters intra-orally and left long for ease of subsequent removal. Note – reconstructive flap is inset after the catheters are secured in place in a standard manner

All patients underwent a partial glossectomy ± appropriate neck dissection (ND) by the surgical ablative team. Prior to reconstruction of the oral tongue defect the surgical reconstructive team and a radiation oncologist assessed the tongue defect and intra-operatively placed afterloading-BRTx Comfort-Catheters (Elekta, Stockholm, Sweden) through hollow stainless-steel catheter guides. Free-hand technique application was used and the catheters were placed parallel to the resection margin, 3-5 mm from the margin itself. The catheters were positioned in a single plane 1–1.5 cm apart and parallel to each other. These were secured in place with a semi-lunar button in contact with the patient’s skin, without the need to suture the catheters in place. The tongue defect was subsequently reconstructed with a local/free flap (Fig. 1). All patients received a tracheostomy.

Following surgery, surgical histopathology was rushed through so as to either remove the BRTx catheters or begin adjuvant BRTx to the tongue within 3–5 days post-operatively. BRTx in the form of high dose rate Iridium^192^ was administered twice daily depending on histopathological findings as follows: ‘Clear’ margins (> 5 mm) no BRTx with catheter removal on the ward; ‘Narrow’ margins (2.1-5 mm) received 34Gy/10Fr; ‘Positive’ margins (≤2 mm) received 40.8Gy/12Fr. BRTx was administered twice daily 6 or more hours apart and over the course of 5–6 days.

Target volumes were defined on a planning CT-scan in ‘in treatment’ position, Clinical Target Volume (CTV) was defined as an expansion of 5 mm around the radio-opaque catheters. The mandible as an Organ at Risk (OAR) was contoured in all cases, no Planning Target Volume (PTV) added. Inverse planning was done using the Oncentra® Brachytherapy planning system (Elekta) with the following constraints to be respected: at least 98% of the prescribed dose covering the target, the volume receiving 150% to be less than 40%, and the point dose on the mandible not to exceed 100% of the prescribed dose. The treatment was delivered on the MicroSelectron® HDR afterloading platform (Elekta). Adequate position of the catheters was confirmed clinically at each treatment with CT planning repeated if there was any change in position secondary to contour changes related to swelling or surgical changes.

External beam radiation to the neck was given to patients with intermediate to high risk criteria on pathology including > 2 positive regional nodes, ENE, and bilateral neck involvement.

For the neck radiation, the neck position at the time of the brachytherapy was reproduced and patient immobilized with thermoplastic mask and custom head rest. The neck was treated with Intensity Modulated Radiotherapy (IMRT) using fixed beam IMRT initially then Volumetric Modulated Arc Therapy for more recent cases (RapidArc, Varian Medical Systems, Palo Alto, California, USA). Target volume and OARs were contoured as per international guidelines [15].

The involved neck was treated to a dose of 55Gy/25Fx, the non-involved neck limited to 50 Gy/ 25Fx. In order to account for overlap with the brachytherapy treatment, the BRTx planning CT scan was fused with the EBRTx planning CT scan and the 50% isodose line delivered by brachytherapy was contoured as an OAR and every attempt was taken to limit the dose to this area to 25Gy maximum dose.

Chemotherapy in the form of Carboplatin 100 mg/m^2^ and Taxol 40 mg/m^2^ given weekly concomitant to radiation was used if there is pathological evidence for extranodal extension (ENE).

As per institutional policy, all patients are followed up in the multidisciplinary head and neck clinic for at least 5 years post treatment completion, with annual chest imaging (chest x-ray or CT) as well as thyroid function tests in patients treated with EBRTx to their neck. Presence, site, and date of recurrence were recorded, as were any treatment related complications using the Radiation Therapy Oncology Group (RTOG) acute/late radiation morbidity scoring schema.

“R” statistical software was used to analyse the gathered data. The Kaplan-Meier method was used to calculate survival and control outcomes, while the Cox regression model was used to assess the influence of various factors in a univariate and multi-variate analysis.

## Results

Between September 2009 and April 2017 55 patients with biopsy proven OTSCC (T1–3, N0–2, M0), median age of 62 (24–92), were recruited into the study and underwent a partial glossectomy ± appropriate ND. Patient and tumour details are provided in Table 1. Fifty three patients had BRTx catheters inserted intra-operatively. Two patients did not undergo BRTx catheter insertion due to their potential proximity to the mandible and hyoid bone, thus increasing the risk of ORN. Of note, these 2 patients had clear margins on subsequent pathological analysis and would have had no BRTx administered according to the study protocol, thus not skewing primary study outcomes.

**Table 1:** Patient Demographics

	Number	%
Male	26	47.3
Female	29	52.7
**pT stage**		
1	27	49.1
2	26	47.3
3	2	3.6
4	0	0
**pN stage**		
0	36	65.4
1	8	14.6
2	11	20.0
**AS/PG**		
I	22	40.0
II	14	25.4
III	8	14.6
IV	11	20.0
**Tumour Differentiation**		
Well	27	49.1
Moderately	21	38.2
Poor	7	12.7

***AS/PG*** **Anatomic Stage/Prognostic Groups**

In total 54 patients were treated with unilateral or bilateral ND (as per primary tumour indications), with one patient avoiding ND for a T1 with less than 3 mm depth of invasion. One patient underwent a delayed/staged ND following primary tumour pathological analysis.

Analysis of primary tumour resection is shown in Table 2. Only 16 patients (29.1%) required and received EBRTx to the neck for ENE/> 2 positive nodes, one of which had ‘clear’ primary resection margins. Thus, 27 patients out of 43 (64.3%) were spared EBRTx all together due to the use of BRTx, and 42 patients were spared EBRTx to the oral cavity or further surgical resection that may otherwise be recommended for ‘narrow’ or ‘positive’ margins. Treatment type administered by overall tumour stage is provided in Table 3.

**Table 2:**
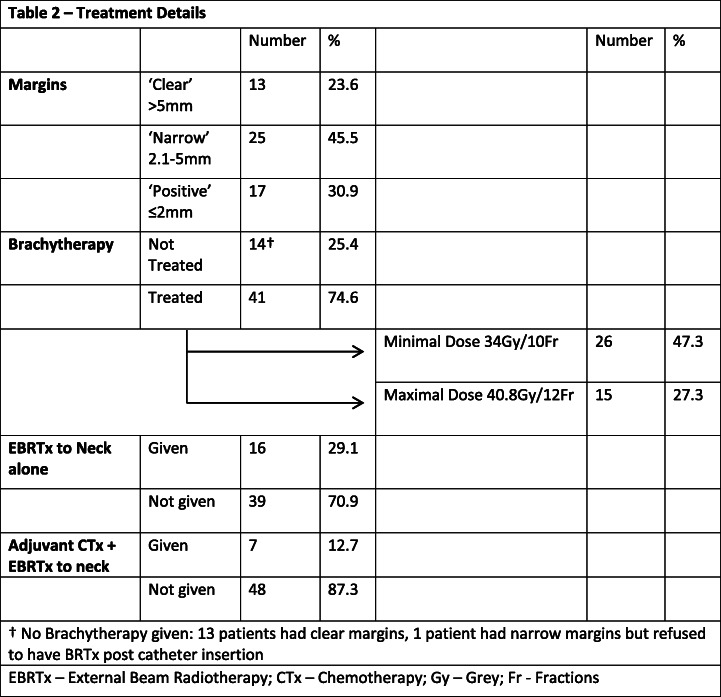
Treatment Details

† No Brachytherapy given: 13 patients had clear margins, 1 patient had narrow margins but refused to have BRTx post catheter insertion

*EBRTx* External Beam Radiotherapy, *CTx* Chemotherapy, *Gy* Grey, *Fr* Fractions

**Table 3:** Treatment by Stage

	Treatment Type			
	Surgery alone	Surgery + BRTx	Surgery + BRTx + CRTx	Surgery + CRTx
**AS/PG Stage**				
**I**	8	14		
**II**	3	10		1
**III**	1	2	5	
**IV**		1	9	1
**Total**	12	27	14	2

*BRTx* Brachytherapy, *CRTx* External Beam Radiotherapy & Chemotherapy, *AS/PG* Anatomic Stage/Prognostic Groups

A total of 12 patients (21.8%) recurred over the median follow up time of 25.4 months (2.9–81.3), with the median time to recurrence being 11 months (6.8–38.8). Further details of observed recurrences are provided in Table 4.

**Table 4:** Recurrence

Median time to Recurrence – 11 months (6.8–38.8)							
				By AS/PG Stage			
	Number	% of all recurrences (*N* = 12)	% of all Patients (*N* = 55)	I	II	III	IV
**Total**	12	100	21.8	2	3	3	4
**Local**	6	50.0	10.9	2	1	1	2
**Isolated Regional**	4	33.3	7.3	0	2	2	0
**Combined Loco-regional**	10	83.3	18.2	2	3	3	2
**Isolated Distant**	2	16.7	3.6	0	0	0	2
**Overall Distant**	4	33.3	7.3	0	0	0	4
**Median Follow up 25.4 months (2.9–81.3)**							

*AS/PG* Anatomic Stage/Prognostic Groups

**Fig. 2:**
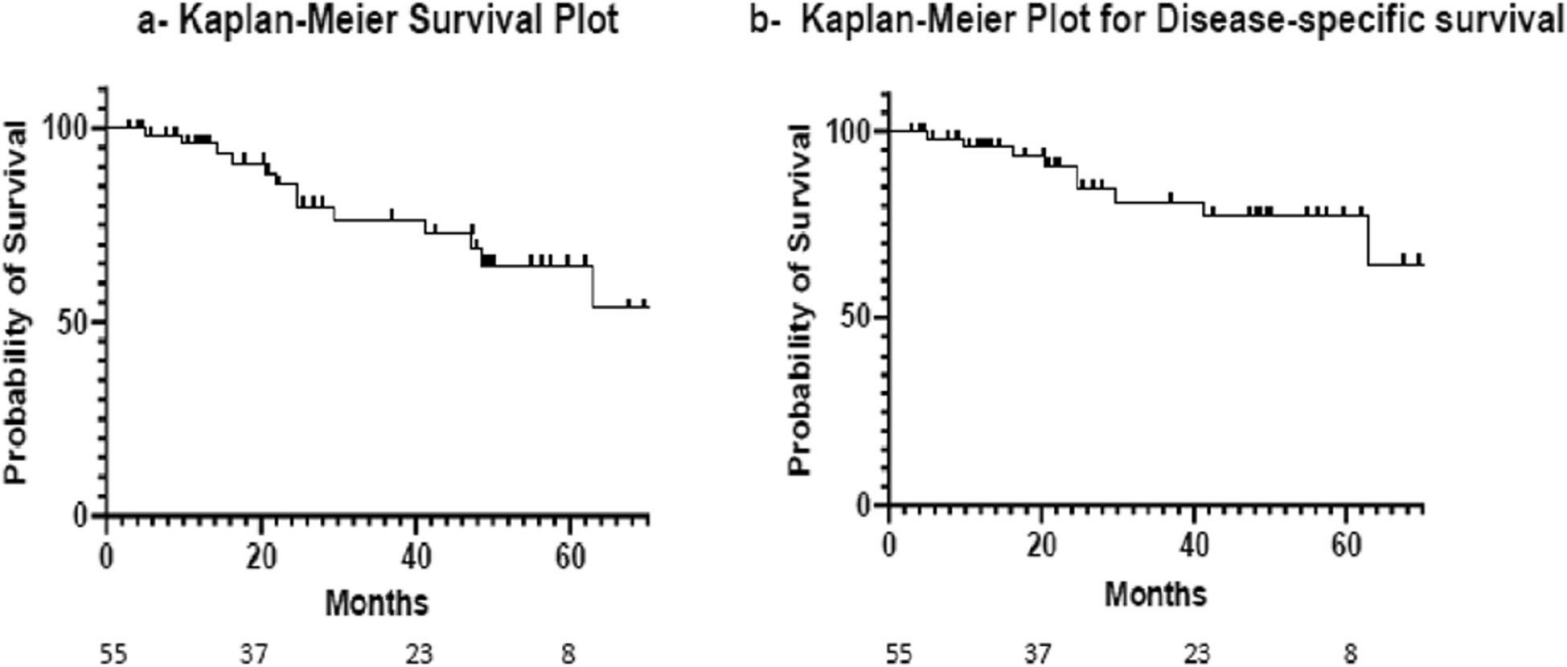
Kaplan-Meier Curves for Overall Survival and Disease Specific Survival. **a** Kaplan-Meier Overall Survival curve. **b** Kaplan-Meier Disease Specific Survival curve

**Fig. 3:**
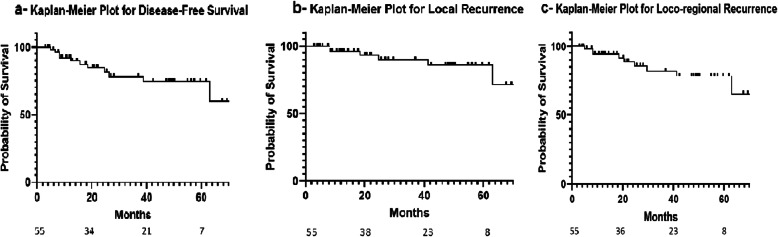
Kaplan-Meier Curves for Disease-Free Survival, Local Recurrence, and Loco-Regional Recurrence. **a** Kaplan-Meier Disease Free Survival curve. **b** Kaplan-Meier Local Recurrence curve. **c** Kaplan-Meier Loco-Regional Recurrence curve

The Kaplan-Meier model revealed an overall survival (OS) of 75.6 and 59.1% at 3 and 5 years respectively while Disease Specific Survival (DSS) was 82.3% at 3 years and 68.6% at 5 years Fig. 2. Disease Free Survival (DFS), Local Control (LC), and Loco-regional Control (LRC) at 3 years were 74.3, 86.3, and 77.7% respectively Fig. 3.

Cox regression analysis failed to show an effect from age, ENE, use of and dose of BRTx, and tumour grade on OS, DSS, LC, LRC. Importantly, margin status was not seen to have an effect on the above various outcomes, likely secondary to the use of BRTx. Multivariate analysis showed a Hazard Ratio (HR) of 4.04 (1.12–14.53) for a primary tumour ≥pT2, nodal disease of pN2 having an HR of 5.25, stage IV disease having a HR of 10.53, Chemoradiation having an HR of 3.54, on Overall Survival. Univariate analysis showed a significant association between pT3 tumour and worse OS (HR 38.05), DSS (HR 31.65), LC (HR 16.68), and LRC (HR 17.72); while pN2 nodal disease was associated with worse OS (HR 5.25) and DSS (HR 4.63). Overall these associations between a more advanced tumour stage and survival and control outcomes have been previously reported and were expected.

There was minimal acute toxicity during the BRTx ± EBRTx. Mild and transient tongue swelling was noted in all patients during BRTx, with the airway being secured with the intra-operative tracheostomy. In 7 patients (12.7%) treated with both EBRTx and CTx grade 3 transient radiation dermatitis was noted. There was a single episode of haemorrhage during the removal of BRTx catheters on the ward at the beginning of the trial, requiring an examination and cautery under general anaesthesia (grade 4).

Late toxicity was noted in 3 patients, characterized by persistent pain localizing to the oral tongue and requiring intermittent opioid-based analgesia (grade 2). Of note, no ORN, dysphagia, or xerostomia requiring salivary substitution was identified over the course of the follow up period.

## Discussion

This study demonstrates the feasibility of using BRTx for margin control in OTSCC. Out of the 55 enrolled patients 53 had the BRTx catheters inserted without difficulty and patients tolerated the catheters well post operatively. The rapid pathological analysis of the resected primary specimen ensured that a decision to administer BRTx and the dose applied could be determined in a timely manner, and treatment was completed within 8–12 days post-surgery. Overall, 25.4% of patients had catheters removed without the need for BRTx administration due to ‘clear’ (> 5 mm) surgical margins. Patient recovery post BRTx catheter removal, whether BRTx administered or not, matched that of patients recovering from partial glossectomy with local/free flap reconstruction in time and complications. Although all patients with BRTx catheters required a tracheostomy for safe airway management, considering that a large proportion of OTSCC require a flap for reconstruction with a tracheostomy, this did not add considerable morbidity to the patient population. All patients were successfully de-cannulated 1–2 days after catheter removal.

This study’s primary outcomes compare favourably with previously published reports on the use of BRTx for margin control in OTSCC +/− EBRTx +/− CTx, as well primary BRTx treatment and ‘traditional’ treatment with surgery followed by EBRTx +/− CTx (Table 5).

**Table 5:** Literature review detailing clinical outcomes of adjuvant post-operative therapy of oral tongue carcinoma. This study’s results (under Ianosvky et al) compares favorably with previously published reports

Paper	TNM	Patient number	Follow up, median (months)	Treatment	OS	DSS	DFS	Local control	Complications - Acute	Complications - Late
**Primary Surgery + Adjuvant BRTx for + ve or Close Margins**										
**Lapeyre et al 2004** [16]	T1–4, N0–3, M0^a^	82	69 (mean)	BRTx^b^ ± EBRTx	68% (5 yr)	80% (5 yr)		81% (2 yr)		43% Grade 1: 12 Grade 2: 17 Grade 3: 8
**Ayukawa et al 2007** [10]	T1–2, N0, M0	28	72	BRTx^b^		96% (5 yr)	92% (5 yr)			8% Grade 1 + 2
**Petera at al 2015** [13]	T1–3, N0, M0	30	40 (mean)	BRTx	73% (3 yrs)		65.4% (3 yrs)	85.4% (3 yrs)	All Grade 2	1 ORN 2 Soft tissue necrosis
**Goineau et al 2015** [9]	T1–2, N0–3^c^	112	80.4	BRTx ± EBRTx neck ± CTx	72% (2 yrs) 56% (5 yrs)	81% (2 yrs) 67% (5 yrs)		79% (2 yrs) 76% (5 yrs)	All grade ≥ 2 mucositis 12% infection requiring ABx.	22% grade ≥ 2 tongue necrosis requiring surgery. 8% pain requiring narcotics
**Ianovski et al 2020**	T1–3,N0–3, M0	55	25.4	BRTx ± EBRTx neck ± CTx	75.6% (3 yrs) 59.1% (5 yrs)	82.3% (3 yrs) 68.6% (5 yrs)	74.3% (3 yrs)	86.3% (3 yrs)	1 haemorrhage (Grade 4)	5% grade 2 pain requiring narcotics
**Primary BRTx ± ND ± EBRTx ± CTx ± Intralesional CTx**^d^										
**Urashima et al 2007** [17]	T1–2, N0, M0	409		BRTx ± EBRTx ± CTx ± intralesional	72.2–82.3% (5 yrs)		56–64.6% (5 yrs)	86–97% (5 yrs)		19.8% ulcer, 6.6% ORN
**Bhalavat et al 2009** [14]	T1–2, N0, M0	57	64	BRTx ± EBRTx	67% (5 yrs)		51% (5 yrs)	59.7% (5 yrs)	BRTx: 47% grade III mucositis. 1 haemorrhage BRTx + EBRTx: 60% grade II, 10% grade III mucositis + skin toxicity	14% submental fibrosis 12.3% soft tissue necrosis 2 ORN
**Akiyama et al 2012** [18]	T1–2, N0, M0	51	45	BRTx	88% (2 yrs)			88% (2 yrs)		9% soft tissue ulcers 9% bone exposure
**Matsumoto et al 2013** [19]	T1–2, N0, M0	67	58.6	BRTx ± EBRTx ± CTx ± intralesional	88.7% (5 yrs)	92.1% (5 yrs)	76% (5 yrs)	94% (5 yrs)		15% grade 3 + 7% grade 2mucositis
**Bansal et al 2016** [20]	T1–2, N0, M0	62	53.5	BRTx ± EBRTx/ND to neck	78.8% (5 yrs)		59.3% (5 yrs)	68.2% (5 yrs)		32.9%: pain +trismus 4.3%, ankyloglossia 7.6%, ORN 1.1%, induration 13.1%
**Primary Surgery ± EBRTx ± CTx**										
**Ling et al 2013** [21]	T1–4, N0–3	210	36.6	Sx +/− EBRTx +/− CTx	44.4% (5 yr)	47.7% (5 yr)				
**Okuyemi et al 2014** [22]	T0–4, N0–3	166		Sx +/− EBRTx +/−? CTx	70.4% (2 yrs) 57.7% (5 yrs)	84.1% (2 yrs) 78% (5 yrs)				
**Mroueh et al 2017** [23]	T1–4, N0–3	325	43 (mean)	Sx +/− EBRTx +/− CTx	61% (5 yrs)	76% (5 yrs)	65% (5 yrs)			
**Liao et all 2017** [24]	T1–4, N0–3	8509		Sx +/− RTx +/− CTx	69% (5 yr)	77% (5 yr)				
**Zhang et al 2017** [25]	T1–4, N0–3	457	39	Sx +/− RTx +/− CTx			68–74.6% (3 yr) 33.1–64.8% (5 yr)α			

^a^ Oral tongue + Floor of mouth subsites

^b^ Low Dose BRTx

^c^ 70% primary tumours, 15% recurrent, 17% 2nd primary

^d^ variable BRTx protocols: including both Low Dose and High Dose Rate treatments, various isotopes (Ra226, Ir192, Au198, Rn222, Cs137), a wide range of dosage (40–70 Gy) and fractionation methods, catheters placed in single and dual planes. Variable rates of surgical management of the neck, and different CTx medications

α grouped into 3 age groups (Young: ≤30, Middle: 46–59, Older: ≥70 years of age)

*OS* Overall Survival, *DSS* Disease Specific Survival, *DFS* Disease Free Survival, *ABx* Antibiotics, *BRTx* Brachytherapy, *EBRTx* External beam radiotherapy, *CTx* Chemotherapy, *RTx* Radiotherapy, *ORN* Osteoradionecrosis, *RTOG* Radiation Therapy Oncology Group

Overall only 8 patients (14.5%) in this study showed evidence of acute complications: 7 patients (12.7%) that were treated with combination of EBRTx + CTx developed transient RTOG Grade 3 radiation dermatitis and 1 patient experienced an episode of haemorrhage from the tongue when BRTx catheters were removed on the ward. Of note, the haemorrhage occurred during the early phase of the trial and was likely related to catheter removal technique, as there were no further complications of this nature with later cases. The bleeding episode was easily managed with cautery that was performed in the operating room under a general anaesthetic. Late complications were only observed in 3 patients (5.4%) and were all limited to ongoing tongue pain requiring opioid medications (RTOG Grade 2). Of note, all 3 patients were treated with surgery and BRTx only, without further adjuvant EBRTx/CTx; two had the maximal dose (40.8 Gy/12Fr) while one had the lower dose (34 Gy/10Fr). It is thus not possible to discern whether it was the initial surgery or the subsequent BRTx that had the greatest contribution to the ongoing opioid analgesia requirements. The study showed no episodes of ORN, dysphagia, or xerostomia requiring salivary substitution. On the whole, the complications profile compares positively with published literature (Table 5).

Statistical analysis of the current findings demonstrates no difference in major outcomes between patients with negative (> 5 mm) margins and positive/narrow (≤5 mm) margins treated with subsequent BRTx. This important finding suggests that BRTx may achieve a ‘sterilization’ effect on the margins, achieving a state where margins have no or limited impact on survival and control outcomes. This is further highlighted by the absence of differences in outcomes between the narrow (2.1-5 mm) and positive (≤2 mm) margins groups. Further consideration needs to be given to these findings in light of the recent paper published by Zanoni et al. in 2017 [26], which proposed margins > 2.2 mm in OTSCC to be considered ‘clear’ after the analysis of 381 cases. Combining the findings of both studies, it is possible to consider future use of high dose BRTx (40.8 Gy/ 12Fr) in patients with ≤2.2 mm margins, sparing BRTx use in surgical margins that are greater.

The use of BRTx for margin control over ‘traditional’ revision surgery +/− EBRTx or EBRTx without further resection has both clinical and logistical advantages. Revision surgery is often made challenging secondary to the local/free flap reconstruction of the tongue and the need for timely operating list planning, potentially placing extra organizational/financial pressures on the surgical unit. Especially if EBRTx is subsequently required, second surgery can also impact the timing of when this can be delivered. Thus, the timing of treatment package may be extended beyond 11 weeks, which has been shown to be associated with worse OS and recurrence free survival in OCSCC [27]. Of note, in the current study 70.9% of patients had their treatment completed within 2 weeks (no EBRTx required), while the use of BRTx did not lead to any instances of EBRTx delay.

**Fig. 4:**
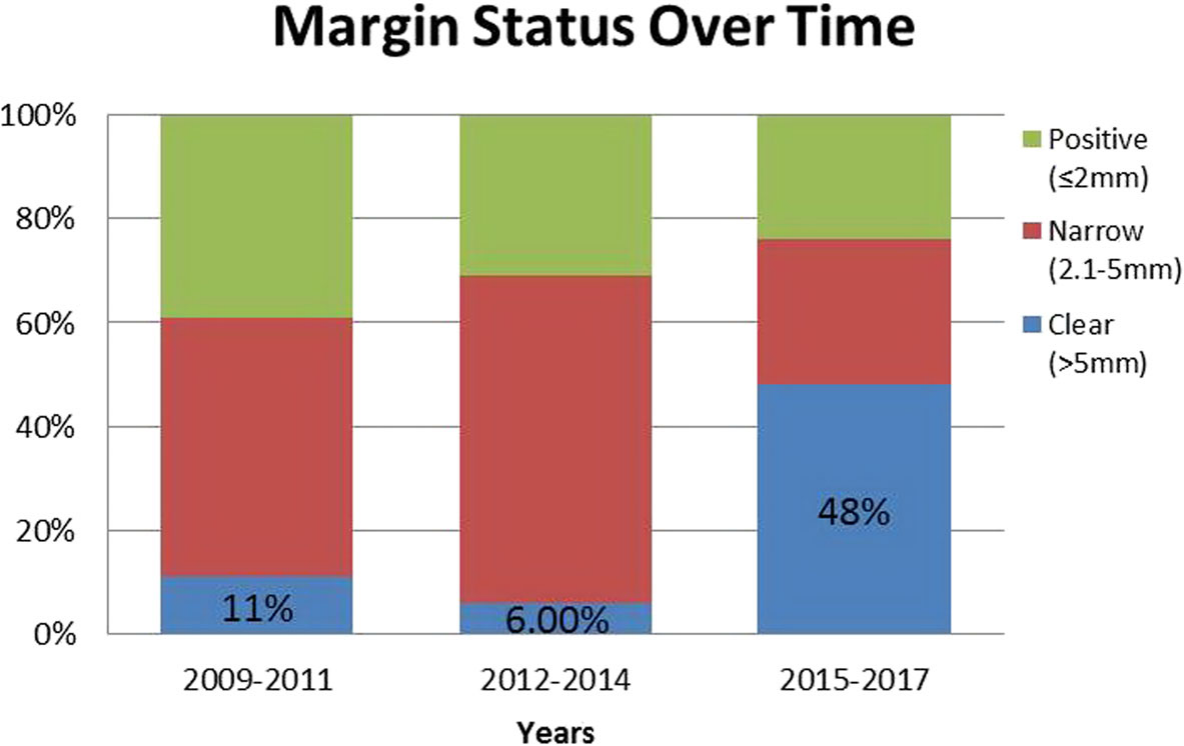
Change in Margin status rates over time

Although surgical margins are an important prognostic factor in OTSCC, these undergo an element of shrinkage by as much as 23.5% within 30 min of resection [28] with limited further shrinkage post formalin fixation [29]. Another contributing factor to margin shrinkage may be the use of monopolar cautery device for the resection of the primary tumour, secondary to thermal injury. During the initial phase of this study needle-point monopolar cautery was used for resection of the primary with 1.5 cm margins marked out after an intra-operative clinical assessment. A high rate of ‘positive’ and ‘narrow’ margins were noted between 2009 and 2011 (89%) and 2012–2014 (94%). As such, in 2015 the resection technique was changed to a cold steel (scalpel blade) excision, after 1.5 cm margins were clinically marked out on the tongue and local anaesthetic (LA) in the form of 1% Xylocaine with Adrenaline was infiltrated. Minimal localized bipolar diathermy was subsequently applied for haemostasis. This technique achieved a significant reduction in ‘positive’ and ‘narrow’ margins to 52% (Fig. 4), as well as proved to be a safe alternative to the classical use of monopolar diathermy with minimal blood loss. The use of cold steel excision with LA is the current practice for glossectomy cases at this institution.

The current study has a number of limitations that benefit from discussion. The absence of a control group in which patients would have undergone ‘traditional’ treatment in the form of surgery followed by re-resection or EBRTx for margin control +/− EBRTx +/− CTx as required is noted. However, it is possible to compare the current outcome measures to the numerous papers published on the success rates of such treatment approach. As such, the treatment algorithm described in this study shows outcomes that are at least as good as the ‘traditional’ treatment approach to OTSCC management as well as the use of primary BRTx, with a significant reduction in both short-term and long-term side effects. The number of patients in the current study is limited to 55, which although is smaller in comparison to most studies published on ‘traditional’ management of OTSCC, compares well to research published on the use of primary as well as adjuvant BRTx in OTSCC and shows clear outcomes that compare favourably to these. It is noted that 96.4% of this study’s population represented T1–2 disease and thus these findings need to be applied with caution to T3 tumours.

It is of note that a more meaningful comparison of outcome should be done with a cohort limited to early T stage since our population is mostly composed of T1–2 cancers. Most of the studies in the literature analyze their outcome by global stage.

However, we did identify two studies by Ling et al. and Mroueh et al. [21, 23] that provide outcome for early T stage oral tongue cancer treated with adjuvant external beam radiation with or without concomitant chemotherapy. These two studies could be used as a comparison group to our cohort.

Ling et al. reported a 3 year disease specific survival of 76.1 and 64.9% in 51 and 75 patients with stage T1 and T2 oral tongue carcinoma respectively. Mroueh et al. analyzed disease recurrence in 176 patients with stage T1–2 oral tongue cancer treated with modern techniques from 2005 to 2009 in Finland. They report a disease recurrence rate of 25 and 29% in stage T1 and T2 oral tongue cancer respectively.

Our results with a 3 year DSS of 82.3% and 3 year LRC of 77.7% compare favorably with this select population of early T stage oral tongue carcinoma.

Similarly, it is important to highlight the exclusion of T4 tumours in the current study (due to the concern for an elevated ORN risk) when comparing primary outcomes with ‘traditional’ treatment research in which these were included. Further work is planned to include higher T-stage tumours in BRTx research, although it is considered that some proportion of T3 tumours and the majority of T4 tumours would not be amenable to BRTx catheter insertion due to proximity of bone and elevated ORN risk. Although the incidence of short-term and long-term side effects was documented, quality of life (QoL) as a whole was not assessed in this patient population. Further investigation into patient-reported QoL measures with BRTx use in OTSCC management will be of important benefit.

## Conclusions

The results of our prospective study suggest that the treatment of OTSCC with partial glossectomy followed by BRTx for positive/narrow margins and +/− EBRTx to the neck +/− CTx achieves outcomes comparable to traditional treatment algorithms. The use of BRTx removes the need for margin re-resection and complex re-reconstruction which may impact treatment package time and apply extra pressure on operative planning, as well as allows the avoidance of EBRTx to the oral cavity and morbidity associated with this. Margin control attained with post-resection BRTx in OTSCC may achieve a state where margins have no or limited impact on outcomes. Administration of BRTx in OTSCC is not difficult and allows acceptable and lower rates of complications than those previously reported with primary and adjuvant BRTx use in OTSCC.

As such, the use of intra-operative brachytherapy is a viable and interesting option to consider in a select population with oral tongue cancer. Our findings would serve as a stepping stone to a more robust multi-institutional trial with direct comparison to standard of practice.

## Data Availability

The data that support the findings of this study are available from the corresponding author upon reasonable request.

## Abbreviations

BRTx: Brachytherapy
CTx: Chemotherapy
CTV: Clinical Target Volume
DFS: Disease Free Survival
DSS: Disease Specific Survival
EBRTx: External Beam Radiation Therapy
ENE: Extranodal Extension
FoM: Floor of Mouth
HR: Hazard Ratio
IMRT: Intensity Modulated Radiotherapy
LA: Local Anaesthetic
LC: Local Control
LRC: Locoregional Control
LVS: Lymphovascular Spread
MDT: Multi-Disciplinary Team
MTB: Multidisciplinary Tumour Board
ND: Neck Dissection
OAR: Organ at Risk
OCSCC: Oral Cavity Squamous Cell Carcinoma
ORN: Osteoradionecrosis
OS: Overall Survival
OTSCC: Oral Tongue Squamous Cell Carcinoma
PTV: Planning Target Volume
QoL: Quality of Life
RTOG: Radiation Therapy Oncology Group
WLE: Wide Local Excision
